# Opportunistic domestic violence screening for pregnant and post-partum women by community based health care providers

**DOI:** 10.1186/s12905-018-0620-2

**Published:** 2018-07-24

**Authors:** Rebecca O’Reilly, Kath Peters

**Affiliations:** School of Nursing and Midwifery, Western Sydney University, Locked Bag 1797, Penrith, NSW 2751 Australia

**Keywords:** Domestic violence, Perinatal women, Health care providers, Screening practices

## Abstract

**Background:**

Domestic violence against women is a global endemic that can commence or escalate during pregnancy and continue postpartum. Pregnant and postpartum women generally access health care providers more at this time than at any other time in their lives. Despite this, little is known about primary health care providers’ screening practices for domestic violence. The purpose of this paper is to present survey findings that identified domestic violence screening practices of community based health care providers in pregnant and postpartum women.

**Methods:**

This paper reports on the survey results of a larger sequential mixed methods study that involved a survey and semi-structured interviews, and used a pragmatic approach to the data collection and analysis. The survey sought information via both fixed choice and open responses. Quantitative data from the surveys were entered into the Statistical Package for Social Science (SPSS™ Version 22) and analysed using descriptive statistics. Open responses were collated and then integrated and presented with the quantitative data.

**Results:**

Results revealed that some health care providers did not screen for domestic violence. Factors contributing to this lack of screening included: a lack of recognition that this was part of their role; and a lack of domestic violence screening policies and/or reminder systems. Further barriers to domestic violence screening were identified as a lack of time, resources and confidence in undertaking the screening and referral of women when domestic violence was detected.

**Conclusions:**

The findings reported in this paper confirm that further insights into the domestic violence screening practices of community based health care providers is required. Findings also have the potential to inform interventions that can be implemented to increase domestic violence screening and promote appropriate referral practices.

**Electronic supplementary material:**

The online version of this article (10.1186/s12905-018-0620-2) contains supplementary material, which is available to authorized users.

## Background

Domestic violence (DV) against women is a global burden occurring in endemic proportions and requires urgent action [1–3]. Domestic violence is also known as Intimate Partner Violence (IPV), or Family and Domestic Violence. While there is no one universal definition there are commonalities within each definition. These include physical, sexual, emotional and psychological abuse, and controlling behaviours [2, 4]. For the purpose of this paper, the term ‘domestic violence’ or ‘DV’ will be used and is defined by the authors as an ongoing pattern of behaviour used to exert power and control to create fear of a current or previous intimate partner through physical, sexual, emotional or psychological abuse, controlling behaviours or a combination of two or more of these behaviours.

A World Health Organisation (WHO) report on studies across 10 countries revealed 13–61% of women had experienced physical violence by a partner; 6–59% reported sexual violence by a partner; and 20–75% reported experiencing one emotionally abusive act, or more, from a partner in their lifetime [2]. Similar statistics have emerged from Australian studies. According to the Australian Bureau of Statistics 16.9% of Australian women have experienced partner violence, specifically physical and sexual violence, since the age of 15 [4]. Furthermore, one in four (25%) Australian women have experienced emotional abuse by a partner since the age of 15 [5, 6].

Pregnancy has been identified as both a potential protective factor and a risk factor for DV, and may be a trigger for violence beginning [7–9]. Australian studies have reported that 5–17% of Australian women experienced DV from an intimate partner for the first time when pregnant and the risk of DV is higher in both pregnancy and the period following birth [9, 10]. Additionally, the World Health Organisation [2] reported that for 8–34% of women in abusive relationships, the violence became worse during pregnancy. There have been further reports that pregnancy is a period of high risk for DV occurring for the first time or escalating during this period [11–13]. As DV is rarely a one off episode, the violence against women may then continue through to the postnatal period [7–9]. Conversely, a cross-sectional study of 768 English-speaking women aged 18–64 years who presented to 2 emergency departments in Ontario, Canada, reported that pregnancy was not a significant risk factor for DV but that prenatal abuse was a predictor of peri- and post-natal abuse [14]. What is consistent in the literature is that the risk of negative consequences for women, the unborn foetus, and infants and children are high when DV occurs during pregnancy or the post-natal period [3, 8, 14, 15].

Whether or not pregnancy and the post-natal period are considered risk factors for DV, these are the times in a woman’s life when she will generally access a health care provider (HCP) more frequently. Therefore, it is an opportune time for HCPs to undertake DV screening [15–18]. Screening for DV is described by the Australian Institute of Health and Welfare [9] as “*a process to identify victims of violence or abuse in order to offer interventions that can lead to beneficial outcome*” (p.vi). The best method of screening for DV, and the benefits of universal screening, is debated. A number of formalised screening tools exist that can be used for DV screening, such as NSW Health Screening for DV tool, Abuse Assessment Screen (AAS), Violence against Women Screen, Index of Spouse Abuse, Danger Assessment Scale, and Conflicts Tactics Scale [17, 19]. Support for formalised tools for DV screening exist with claims that such screening instruments can accurately identify women experiencing DV [20, 21]. However, there is literature that has raised questions of the validity of some formal DV screening tools due to inadequate testing of such tools [17, 22, 23].

While the increase in identification of DV towards women from screening is not denied, there are questions as to the health benefits to women from such identification [3, 14, 24]. The World Health Organisation have recommended that, rather than implementing universal screening for DV, screening women who present with conditions that may be caused or complicated by DV is preferable, but only if such screening is also offered alongside appropriate post-screening action [18]. Other research also supports that DV screening is only beneficial if accompanied by interventions that can support the victim/s of the abuse [25, 26].

Research to date indicates that women are not being routinely screened for DV by all HCPs [27, 28]. The reluctance of HCPs to screen for DV is purported to be due to inadequate knowledge and training with DV screening tools and the belief that resources to support identified victims are inadequate [27, 28]. Furthermore, HCPs’ lack of DV screening has been identified as due to fear of offending patients, a lack of time and forgetting to routinely screen, a lack of patient privacy, workload issues, reluctance to see DV as a health issue, and HCPs’ personal discomfort with DV [27–29]. GPs have also reported reluctance in initiating DV screening due to a lack of formal training and appropriate referral pathways in the event of a positive screen [28, 29].

Midwives have a central role in the provision of maternity care to women which place them in a prime positon to screen for DV [30]. In addition to barriers described by other health professionals, midwives have reported barriers to screening to be the personal nature of the topic, negative ideas about victims of abuse, language barriers, and the woman’s partner being present during care [30–32]. Consequently, international studies have highlighted that midwives feel inadequately prepared to support women who disclose DV [29, 33].

Despite the reluctance of HCPs to screen for DV, research has found that women readily accept being asked about DV by HCPs [18, 31, 34]. Additionally, routine screening for DV has been recognised as an important identification and preventive method that allows the implementation of early intervention strategies for women exposed to DV [17, 35, 36]. In 2003, NSW Health released a DV policy that enforced mandatory routine DV screening of all women that attend public antenatal and early childhood services in NSW, Australia. This was followed by a directive that all public antenatal, early childhood health, drug and alcohol and mental health services introduce the NSW Health Domestic Violence Routine Screening Program [37]. The same policy highly recommended private and not-for profit community based health care services introduce routine DV screening into their practices [36]. Despite this, there are no universally accepted guidelines for, or consistent approaches to, health care provider practices in DV screening across NSW or Australia.

The lack of consistency in the approach to DV screening practices across Australia may be attributed to each Australian State and Territory having their own Government structures. However, there have been numerous Australian government strategies developed to address DV against women; for example, ‘Time for Action: The National Council’s Plan for Australia to reduce Violence against Women and their Children, 2009-2021’; the Third Action Plan 2016–2019 of the National Plan to Reduce Violence against Women and their Children 2010–2022; and ‘Stop the Violence, End the Silence’ [38, 39]. In an effort to seek consistency, significant policy, structures and investments by all stakeholders in DV prevention and intervention across Australian State and Territory Government bodies are coordinated by the Council of Australian Governments (COAG). Despite multiple strategies being implemented, under-reporting of DV persists and there is little known about the screening practices of community HCPs in NSW, Australia. The aim of this research was to explore DV screening practices of community based HCPs.

## Methods

This paper presents the survey data from a mixed methods study and provides insights into the DV screening practices of community HCPs in one area of NSW, Australia.

Prior to recruitment and data collection, the study was approved by Western Sydney University Human Research Ethics Committee (Ethics Approval no. H11294).

### Sampling & Recruitment

A convenience sample of community HCPs was recruited for this study. Inclusion criteria were that participants needed to be employed as a community HCP who provided care to pregnant or post-natal women in Western Sydney Local Health District. Specifically, participants were General Practitioners (GPs), private practice Midwives (PPM), and Registered Nurses (RNs) either from Women’s Health Care centres or General Practice Surgeries. This Local Health District was chosen due to the high prevalence of low income families, unemployment, low educational attainment, Aboriginal origin, women with disabilities and women from culturally and linguistically diverse backgrounds [40] which are all known DV related risk factors [41]. Initially, an introductory letter and an electronic link to the survey were distributed via an email list from a medical publishing company. Hard copies of surveys were also personally distributed by the research team to individual general practice clinics due to a poor response to the electronic survey.

### The survey

The survey was developed by consulting published DV screening literature and experts. Face and content validity was gained by distributing the initial survey to a panel of 10 academics whom have expertise in survey development or who were still practicing clinically as RNs or midwives. Feedback was considered by the 2 authors and modifications were made prior to implementation in the study. The final survey was comprised of 20 fixed-choice questions. There were 11 questions that asked about DV screening practices and 9 demographic questions. Of the 11 questions that asked about screening practices, 5 of these questions asked for additional open-ended responses. Survey questions are shown in more detail in Additional file 1.

### Study design and data collection

The larger study that the survey sits within was a sequential mixed methods study. Underpinning this sequential mixed method study was a pragmatic approach as the researchers used the methods of data collection and analysis that would best answer the research question [42, 43]. Pragmatism as a research method aligns with empirical enquiry that allows for solving practical problems in the “real world” while being guided primarily by the researcher’s desire to produce socially useful knowledge [44]. As such, it is well suited to understanding the social health issue of primary health care practitioners DV screening practices.

The research question was “What are the practices of community based health care providers (HCPs) in screening peri-natal women for domestic violence?” The survey employed a descriptive design and integrated data from both fixed-choice and open response questions to provide greater context and insights into the DV screening practices of community HCPs.

### Data analysis

Quantitative survey data were entered into the Statistical Package for Social Science (SPSS Version 22) and analysed using descriptive statistics. Frequencies and percentages of data obtained from the survey were determined. The open ended responses were grouped according to respective questions on the survey and were integrated with the descriptive quantitative results [45, 46].

## Results

The survey was completed by 48 community based, non-government health care providers. Of the 48 respondents, 33 were GPs, two were PPMs, ten were RNs, and three did not indicate their specific profession. Fifteen of the respondents were men (all GPs) and 26 were women. Seven respondents did not answer the question on gender. Qualifications of respondents included PhD (*n* = 2, 4.2%), Postgraduate degree (*n* = 6; 12.5%), Royal Australian College of General Practitioners fellowship (*n* = 2; 4.2%); Bachelor’s degree (*n* = 29; 60.4%), Hospital certificate (*n* = 1, 2%), Graduate certificate (*n* = 1, 2%) and Graduate diploma (*n* = 1, 2%). Six respondents (12.5%) did not provide highest qualifications. Respondents had been qualified for between 1 and 52 years and most (*n* = 29; 60.4%) were employed on a full-time basis. As well as providing insights into their DV screening practices, respondents highlighted barriers to screening.

### Workplace policy and reminder systems

When asked about the existence of workplace policies and reminder systems related to DV screening, 23 (48%) respondents indicated they were unsure whether policies existed, and 17 (35%) stated there were no policies. Only 7 (15%) reported that their workplace had a policy for DV screening and one respondent did not answer. Of the 48 respondents, only 3 (6%) reported having a reminder system for DV screening in their workplace, 27 (56%) reported no reminder system and 16 (33%) indicated they were unsure if reminder systems were in place. Two (4%) respondents did not answer this question.

### Screening practices

#### Do you screen?

Eighteen (37.5%) of the 48 respondents reported not screening for DV in the 6 months prior to completing the survey. While 28 (58%) reported screening for DV, 17 (35%) indicated they used general questioning rather than formalised DV screening tools. Nineteen (40%) reported having screened between 1 and 5 women, and only 9 (19%) participants had screened more than 5 women in the prior 6 months. See Table 1 for a breakdown of screening practices according to employment status of respondents.

**Table 1:** How many women screened in the last 6 months?

Employment status	How many antenatal and/or postnatal women have you screened for domestic violence in the last 6 months?				Total
	0	1–5	> 5	Not answered	
GP	14	14	5	0	33 (69%)
PPM	0	1	1	0	2 (4%)
RN	3	3	3	1	10 (21%)
Not stated	1	1	0	1	3 (6%)
Total	18 (37.5%)	19 (39.5/%)	9 (19%)	2 (4%)	48 (100%)

### When do you screen?

There were 23 open-ended responses related to when screening for DV was undertaken. Nine respondents reported they screened perinatal women for DV routinely. One participant screened all women on their first appointment and one reported “*I screen everyone I see*”. The remaining respondents reported screening during the initial antenatal visit only, or both the antenatal and postnatal visits.

Twelve respondents identified that they screened for DV when women presented with signs they considered to be ‘red flags’. These ‘red flags’ included reports or observation of anxiety, depression or stress, signs of physical injury and information gained from the woman’s medical history or personal account. One respondent reported they did not screen and another indicated they screened *‘Opportunistically at check ups’*.

### Barriers to screening

Twenty-five participants provided a range of reasons for not screening for DV in the open responses. Among their reasons 4 respondents reported DV screening did not fall within their usual care practice stating *“I don’t routinely screen - only if there are questions around social support or mental health”*; “*Not part of usual practice but not against the idea”* and *“Not part of my routine screening”.* Six respondents indicated they believed someone else would screen with statements such as “I *assumed they [women] were screened at booking in visit at hospital*”, “*Most antenatal clinic screen for it and I refer all my patients to antenatal clinic*” and “*The doctors do it*.”

Another reason identified by a number of respondents (*n* = 6) was that they felt the resources available to facilitate the DV screening process were inadequate. Resources required to support the HCP were identified as education on how to screen for DV and the availability of screening tools. This was evident in statements such as: *“Not enough education”; “Insufficient screening tools”* and *“No preset screening tool. No education on how to”*.

Relying on women to reveal they were experiencing DV was reported by 4 respondents. Statements that represented this reliance on women to divulge DV included: *“High levels of violence in the community in which I work but generally women come forward when they want to change their situation”; “I would hope the patient would open up to me”;* and *“No patient has complained about Domestic Violence”.* Other respondents identified time constraints as a barrier to DV screening citing *“Too much to get through in the screening*”, and “*Time restrictions – but always screen for depression”*. A further 3 responses indicated that DV screening was never considered during consultations. These respondents provided the following explanations: “*Never thought about screening them”, “Forgetfulness” and “I don’t do it because I just don’t think of it”.*

#### Training

Just over half of respondents (*n* = 26; 54%) had not had any DV specific training. Eighteen (38%) had received some type of training however only 13 of these found the training useful. The majority of those who had training reported this to be self-taught or via in-service education.

Thirteen respondents provided written responses related to training for DV screening. Within these responses, several respondents (*n* = 8) revealed that they had not completed training for DV screening as it was not accessible to them. Examples of responses portraying access issues included being *“Unsure where to get training”*, *“As I work outside the system, it is more difficult to access”, “Haven’t had the opportunity to have specific training”* and *“Time constraints”.* One respondent had *“Not [had] specific training about screening but had training on what to do if it [DV] is discovered”.*

Two respondents indicated they did not find training for DV screening useful because it was *“Too brief”, “Not detailed enough”,* and they *“Can’t specifically recall it”.* Two of the open responses indicated that respondents had not undertaken training as *“Domestic violence is not prevalent in our practice”*, and DV was *“Not really my area”.*

#### Confidence

The survey used a likert scale to measure respondents’ confidence in undertaking DV screening. Response selections ranged from ‘not at all confident’ to ‘very confident’. Only 12% of respondents who had not undergone training for DV screening were ‘very confident’ compared to 28% of respondents who reported having had training. Similarly 35% of respondents who had not undergone training for DV screening reported being ‘not very confident’ compared to 11% of respondents who had training in DV screening.

There were 15 open responses that related to how confident the HCP’s were in undertaking aspects of DV screening. One third (*n* = 5) of these responses alluded to them feeling concerned about what to do with a positive DV screen. For example, as some respondents explained *“I am confident in asking [about DV] and assessing however unsure of what to do if the situation occurs”;* ***“****Unsure who to report to”* and *“Once DV is discovered, directing women to the appropriate services”.* A further 5 respondents expressed they were least confident in encouraging the women to divulge DV. Examples of quotes that represent this are: not confident in *“Breaking the ice”* and that it was a *“Difficult subject for some patients to communicate about”.* Several respondents (*n* = 4) conveyed their lack of confidence in DV screening was due to not having adequate training and access to standardised procedures and screening tools. These concerns are represented in the following quotes where respondents indicated there was *“No obvious protocol”* and they required *“A standardised screening scale”* and *“more training and skills not to miss any important information”.* The remaining respondent revealed a lack of confidence in screening for DV due to concern about the *“Severity of stories heard”.*

### Finding evidence of DV and referral

Additional to enquiring about screening practices, the survey also asked whether participants who had screened had found evidence of DV. Twenty-nine (60%) confirmed that they had and referred women to DV specific services. Although 18 (38%) respondents stated they had not found evidence of DV after screening, 8 of these reported they had referred a DV victim to DV specific services.

A variety of options were provided on the survey for respondents to indicate which services they referred women to when DV was identified. Respondents could select more than one service. The most popular DV referral services reported by respondents were psychologists (*n =* 16) and counsellors (*n =* 16), closely followed by women’s refuges *(n =* 13). Services less commonly referred to were police (*n =* 10), community services *(n =* 9), legal services (*n =* 6) and social workers (*n =* 3).

There were 16 open responses completed regarding the responses of the HCPs to a positive screen for DV. Most of these responses indicated the HCPs used multiple strategies to assist the women. These strategies included counselling the women (*n* = 4), and referred them to specialist health care practitioners (*n* = 9) and women’s refuges (*n* = 2). Three respondents noted that they encouraged the women to contact police and 4 specifically mentioned that they ensured the women were safe. Two respondents mentioned follow up for the women. Other strategies included listening to the women and allowing them to vent and providing educative brochures. One respondent reported providing DV helpline information for a woman who stated she did not want assistance.

## Discussion

This survey examined DV screening practices of community based HCPs, specifically GPs, PPMs and RNs, for pregnant and postnatal women. As well as highlighting practices of DV screening, findings from this study provided insights into barriers to such screening and the needs of HCPs in order to encourage DV screening and referral. While some responses from the survey add to existing knowledge of DV screening practices, barriers, and referral, there were some results that are not prominent in existing literature.

Results from this study showed that just over half of respondents (58%) screened perinatal women for DV. This meant that 42% did not screen, which is consistent with literature that reports a large number of HCPs do not screen for DV [27–29]. Of particular note in this current study were those respondents who did screen for DV favoured a more generalised approach to DV screening rather than using formalised tools. This may be in part due to the fact that the validity of formalised screening tools is rarely tested [17, 22, 23]. Further testing of the reliability and validity of DV screening tools is necessary before HCPs can be expected to use such tools in their daily practices. Further investigation into the DV screening practices community HCPs choose is warranted and this will be explored in the interviews in the second phase of this study.

Additional to the lack of validation of DV screening tools, there is some debate regarding the effectiveness of DV screening and its place in HCPs daily practices. However, there is consensus that perinatal women should be screened for DV as long as this is supported by appropriate interventions and resources for victims [22–24, 34]. Further, research has highlighted the importance of training that assists HCPs with identifying signs and symptoms of DV and informs the referral of victims to effective services [22]. Results from the current study identified that many HCPs had not undertaken formalised training for DV screening and that they lacked confidence and knowledge in intervention and referral processes. Specifically, inadequate knowledge of supportive resources available for victims of DV, or the lack of such resources, was a barrier to respondents undertaking screening. This is an important finding and adds support to a growing body of evidence that DV screening should be accompanied by adequate referral resources [28, 29, 47–52].

Results from this study identified additional barriers that are not widely reported in the literature. In particular, some HCPs did not perceive DV screening to be a part of routine care, while others presumed screening would be undertaken by someone else. There was also the assumption by some HCPs that women who were experiencing DV would self-disclose. Each of these barriers to screening warrants deeper exploration. Education is required to highlight how all HCPs can contribute to supporting and ensuring the safety of women and children experiencing DV. Further, the belief that women will self-disclose without being asked must be dispelled. As shown in previous research, women are unlikely to self-disclose DV unless asked directly and do find it acceptable for their HCP to ask about DV [18, 31, 34].

An important and under reported barrier to DV screening identified in this study was a lack of DV screening policy and reminder systems within the respondents’ work places. This finding is disheartening given the multiple Australian Government strategies in place to support DV identification and intervention policies [38], and in particular the NSW Health DV policy [37]. Additionally, the WHO has issued guidelines to support policy-makers and those responsible for planning, funding and implementing health services and professional training in the areas of medicine, nursing and public health [18]. It is clear that policy implementation for DV screening and referral across all health care sectors needs to be reviewed.

A number of the HCPs who participated in this study reported having experienced positive DV screening results. In these instances, referral was made predominantly to psychologists. Referral to any services is a better option than no referral at all; however, this as a common option raises the question of attitudes towards victims of DV needing mental health care as a priority over protection from the perpetrator. While counselling is identified as an appropriate intervention for victims of DV, HCPs must ensure the intervention and referral processes are immediate, holistic and individualized to the victim’s needs [18, 22, 23, 53]. Existing literature identifies that DV referral and intervention is multi-layered and must begin with immediate, first-line support that assesses further resources required for appropriate referral [18, 22, 23, 53]. Obvious in the findings from this study is the need for education for HCPs that addresses individualised, holistic approaches to appropriate and timely intervention for the victim.

### Limitations

Due to the small sample size, results from this study need to be interpreted with some caution. However, the study provided some new insights into the DV screening practices of community HCPs and some of the results are strongly supported by previous research. While this study has reported on overall screening practices and barriers to screening, it has not identified what facilitates community HCPs to conduct DV screening. A further limitation of the study may be the skew in favour of a higher number of GP respondents compared to RNs and PPMs. However, the ratios of the various disciplines were representative of the overall study population.

### Recommendations

The pragmatic methodology resulted in findings that allow for the development and implementation of practical interventions to enhance DV screening practices. Previous research supports the need for all HCPs who provide care to pregnant and postnatal women to be well educated and trained in DV screening and appropriate referral [28, 30, 31, 52]. The current study, like others, indicates this is not occurring despite recommendation by Government bodies and the WHO [18, 37, 38]. A recommendation from this study goes beyond stating there is a need for education and training for all HCPs who provide health care to women. The authors recommend that further research be undertaken to determine the best means of developing and delivering DV screening education and training to ensure it meets the needs of all multi-disciplinary HCPs. This is integral to ensure HCPs identify and provide appropriate support and referral for women and children experiencing DV.

In NSW there are limited resources for training which means that existing services are stretched beyond their limits. This calls into question the level of commitment underpinning the Australian Government rhetoric in addressing integral aspects of DV including the educational requirements of the front line HCPs. Implementation of compulsory DV education into undergraduate curricula of health care disciplines is recommended. Further, to ensure contemporary, evidence based practices are adopted, mandatory education for DV screening for all practicing HCPs could be undertaken as a requirement of registration renewal. Such strategies however, require the support of both regulatory and Government bodies.

Non-government organisations that provide health care to pregnant and postnatal women should have a mandatory DV screening policy in place that encompasses the best method for implementing DV screening. In NSW, Australia, mandatory screening by Government funded Area Health Services and a standardized screening tool already exists [19, 36]; however, the uptake of such tools in community health care settings is limited. This may be due to a lack of awareness or disagreement with the approach of screening. It is recommended that current screening tools be adapted to the primary health care setting. Furthermore, a guide for referral practices for women who have a positive screening result is required to increase screening rates as well as provide appropriate support for women and children affected by DV.

## Conclusions

Health care providers often face significant challenges in screening women who are pregnant or post-partum for DV. These challenges can stem from both internal and external sources. Regardless of the source of challenges, health care workers need to be supported and encouraged to undertake regular and suitable education and training for DV screening and referral when indicated, to ensure they are providing holistic and responsible care to their patients. Overcoming barriers to screening pregnant and postpartum women is crucial as the responsibility of care extends to unborn and living children.

## Additional file

Additional file 1: Study survey of all fixed response and open ended questions. (PDF 62 kb)

## Abbreviations

DV: Domestic violence
GP: General Practitioner
HCP: Health Care Provider
PPM: Privately Practicing Midwife
RN: Registered Nurse
