# Supplementary material for: Opportunistic domestic violence screening for pregnant and post-partum women by community based health care providers
Source: BMC Womens Health. 2018 Jul 24;18:128. doi: 10.1186/s12905-018-0620-2 (PMC6056948; doi:10.1186/s12905-018-0620-2)
Supplement: Supplementary file 1 — Study survey of all fixed response and open ended questions. (PDF 62 kb) [file 12905_2018_620_MOESM1_ESM.pdf]

WESTERN SYDNEY
UNIVERSITY

Opportunistic Screening for Domestic Violence by Community Health Care Providers of Antenatal and Postnatal Women

1. Have you read and understood the participant information sheet?

Yes / No (Please circle correct answer)

2. Are you involved in the provision of care for antenatal and/or postnatal women?

Yes / No (Please circle correct answer)

If no – thank you for time. You are not required to answer any further questions.

3. Which of the following best reflects your place of employment?

General Practice	1
Medical Centre	2
Private Practice	3
Independent Practitioner	4
Other (please specify)	5

____

4. Do you screen antenatal and postnatal women for domestic violence?

Yes / No (Please circle correct answer)

If your answer to question 3 is No, is there a particular reason why not?

____

____

____

5. How often do you screen antenatal and postnatal women for domestic violence?

Never	Rarely	Sometimes	Frequently	Always

6. Have you undertaken specific education and training on implementing domestic violence screening and responding appropriately?

Yes / No (Please circle correct answer)

If yes to question 6, where did you receive training?

NSW Health training	1
In-service training	2
Tertiary education	3
Post graduate education	4
Self-taught	5
Other (please specify)	6

___

If yes to question 6, did you find the training useful?

Yes / No (Please circle correct answer)

Please elaborate.

___
___
___

If no to question 6, is there a particular reason that you haven't received training?

___
___
___

7. How confident are you in undertaking screening for domestic violence?

Not at all Confident	Not Very Confident	Unsure	Slightly Confident	Very Confident

If your response was 'Very Confident' please move to Question 8; otherwise, please elaborate on which aspects you are not confident with

___
___
___

8. How many antenatal or postnatal women have you screened for domestic violence in the last 6 months? (Please ✓ *one only*)

0 ☐ 0   1-5 ☐ 1   6-10 ☐ 2   11-15 ☐ 3   16-20 ☐ 4   >20 ☐ 5

9. How many times do you screen each antenatal or postnatal woman? (Please ✓ *one only*)

1 ☐ 1   2 ☐ 2   3 ☐ 3   4 ☐ 4   >4 ☐ 5

10. When do you screen antenatal or postnatal women for Domestic violence?

____

____

____

11. What domestic violence screening tools do you use? (Please ✓ *all that apply*)

NSW Health Screening for DV tool	1
Abuse Assessment Screen (AAS)	2
Violence Against Women Screen	3
Danger Assessment Scale	4
Index of Spouse Abuse	5
Conflicts Tactics Scale	6
Other (please specify)	7

____

12. Does your facility have a reminder system for domestic violence screening?

Yes / No (Please circle correct answer)

13. Have you ever screened and found evidence of domestic violence?

Yes / No (Please circle correct answer)

If Yes: How did you respond?

____

____

____

14. Have you ever referred a victim to any resources specific to domestic violence?

Yes / No (Please circle correct answer)

If yes, which resources did you refer to? (Please ✓ *all that apply*)

Legal Services	1
Police	2
Women's Refuge	3
Counselling Services	4
Psychiatrist	5
Psychologist	6
Community Services	7
Social Worker	8
Other (please specify)	9

____

15. Are you:

Male / Female (Please circle correct answer)

16. What is your current age? _____ years

17. What is your highest educational qualification? (Please ✓ one only)

Doctorate	1
Masters	2
Bachelor's Degree	3
Graduate Diploma	4
Associate Diploma	5
Graduate Certificate	6
Hospital Certificate	7
Other (Please specify)	8

____

18. Under what classification are you currently employed? (Please ✓ *one only*)

General Practitioner	1
Independent Midwife	2
Registered Nurse	3
Enrolled Nurse	4
Other (Please specify)	5

____

WESTERN SYDNEY UNIVERSITY

19. In what year did you first qualify? ______________________

20. Are you employed full-time or part-time in your current role? (Please ✓ *one only*)

Full-time ☐ Part-time ☐

21. How many hours per week are you employed?

___________ (Hrs/week)

22. In what postcode area is the main facility in which you work? ____________

Thankyou very much for taking the time to complete this survey.
Your input is valuable and we appreciate your participation.

The research team are very interested in your experiences of domestic violence screening specific to antenatal and postnatal women. If you would be willing to share your experiences and participate in an interview please contact:
Dr Rebecca O'Reilly via re.oreilly@westernsydney.edu.au
OR
(02) 9685 9490

For information on DV screening and referral training please visit the NSW Health Education Centre Against Violence website (see link below) OR email Dr Rebecca O'Reilly for an electronic copy of ECAVs Courses and Publication Book

http://www.ecav.health.nsw.gov.au/online-shop/courses/domestic-violence/
